# A Turning Point in Typhoid Control

**DOI:** 10.1093/infdis/jiy417

**Published:** 2018-09-05

**Authors:** Adwoa D Bentsi-Enchill, Andrew J Pollard

**Affiliations:** 1 Department of Immunization, Vaccines and Biologicals, World Health Organization, Geneva, Switzerland; 2 Oxford Vaccine Group, Department of Paediatrics, University of Oxford, United Kingdom; 3 National Institute for Health Research Oxford Biomedical Research Centre, United Kingdom

**Keywords:** enteric fever, paratyphoid, *Salmonella*, surveillance, typhoid

The highest burden of morbidity and mortality associated with *Salmonella enterica* serovar Typhi and *S. enterica* serovar Paratyphi A occurs in South and Southeast Asia and in sub-Saharan Africa. In the last 2 decades, significant contributions to our overall understanding of typhoid fever and paratyphoid fever (collectively enteric fever) have been achieved through key population-based disease burden studies. Of particular note, the landmark Diseases of the Most Impoverished (DOMI) project, conducted between 2000 and 2008, documented the high incidence of blood culture–confirmed typhoid and several epidemiological aspects of the disease in 7 Asian countries [1]. Similarly, the Typhoid Surveillance in Africa Program, conducted in 13 countries in sub-Saharan Africa between 2010 and 2014, generated significant data to fill the knowledge gaps on typhoid fever and nontyphoidal *Salmonella* disease in that geographic region [2]. Despite these large multicountry studies, and other single-country studies published in recent decades, several epidemiological gaps remain. Several current population studies are anticipated to add to the growing body of knowledge on all 3 invasive *Salmonella* diseases that will make major contributions to their effective control [3–6].

In this supplement, the Surveillance for Enteric Fever in Asia Project (SEAP), a multicountry study covering Bangladesh, India, Nepal, and Pakistan, reports data from a retrospective records review of enteric fever in Phase I of the study [3]. The site-specific findings reported in this supplement [7–10] confirm that enteric fever remains an important public health burden in the region a decade after the DOMI study. These SEAP Phase I reports and, importantly, future results from the prospective studies in Phase II will be invaluable in guiding control strategies for typhoid and paratyphoid fever in Asia. The experience in Bangladesh illustrates opportunities for leveraging existing surveillance approaches and resources where possible [8].

Andrews et al report a low blood culture positivity rate of 4.1% among clinically diagnosed enteric fever cases [7], reinforcing the poor reliability of clinical diagnosis and, conversely, the importance of strengthening surveillance for blood culture confirmation in all typhoid- and paratyphoid-endemic countries. Furthermore, Antillon et al in their systematic review report a blood culture diagnostic sensitivity of 0.59 (95% confidence interval, .54–.64) and found a significant “but modest” relationship between blood volume and blood culture sensitivity, irrespective of patient age, prior antimicrobial use, or other potential confounders [11]. These reports are an important addition to the understanding of diagnostic sensitivity and will allow key adjustments to be made in enteric fever burden estimation.

All participating sites report data on antimicrobial resistance, which are key considerations for countries conducting enteric fever surveillance given the alarming trends in antibiotic resistance, including the recent discovery of an extensively drug-resistant (XDR) *Salmonella* Typhi strain that caused a typhoid fever outbreak in Sindh Province, Pakistan. The emergence of that XDR strain, which has been described as encoding resistance to all the major antimicrobials that have been routinely used for treating typhoid fever over the last 7 decades, amplifies the urgent concerns about narrowing therapeutic options for treatment and control of typhoid fever, and a real need for culture and antimicrobial sensitivity to guide treatment of typhoid fever [12, 13].

Kaljee et al provide critical insights on community and patient perceptions and other socioeconomic factors underlying healthcare utilization patterns in Nepal [14]. We encourage typhoid researchers and endemic countries to gather such data as an important contribution to interpreting epidemiological data and designing effective control strategies. In his supplement article, Luby presents a compelling discussion on the challenges of short-term to medium-term strategies for typhoid control focused on improvements in water and sanitation and the potential value of combined vaccination and water, sanitation and hygiene (WASH) strategies [15]. The data on access to “improved water” are imperfect in many countries and there are pitfalls in what is counted as improved water. However, what is indisputable is the potential benefit from WASH for enteric fever and beyond.

Collectively the SEAP Phase I data help to fill the knowledge gaps in Asia that existed despite the valuable data generated from the DOMI project and other key studies in the last 2 decades. The World Health Organization (WHO) recently issued recommendations for the programmatic use of typhoid conjugate vaccine (TCV) for the control of typhoid fever in both endemic and epidemic settings [16], supported by data from the prototype conjugate vaccine trialled in Vietnam [17], immunogenicity studies [18] with the current generation of TCVs, and efficacy demonstrated in a human challenge model [19]. With the first WHO prequalification of a TCV [20] and a decision by Gavi to support eligible countries for TCV introduction [21], the first countries are set to make decisions on the routine use of vaccination for typhoid control. These important findings from SEAP and other ongoing burden studies will inform policymakers’ decisions on the TCV delivery strategies and typhoid control more broadly. To that end, the supplement article by Lo et al [22] adds to the existing and emerging data based on modeling studies on the cost-effectiveness of different vaccination strategies and the estimated impact of vaccination. These global studies do not necessarily provide a country perspective and further cost-effectiveness analyses by countries will no doubt be needed, perhaps more so for countries that will be graduating from Gavi support in the near term. Field implementation studies in Africa and Asia through the Typhoid Vaccine Acceleration Consortium [23], a demonstration project in Navi Mumbai, India [24], and resistant-typhoid control efforts in Sindh Province, Pakistan [25], will provide critical momentum to support wider implementation.

Another limitation of SEAP and the other burden studies is that the use of sentinel sites does not permit full understanding of the heterogeneity of typhoid and paratyphoid fever across large geographic regions and especially between urban and rural settings. This reemphasizes the critical need for investments in good-quality prospective surveillance in endemic countries. Blood culture is the mainstay for the confirmation of enteric fever for the foreseeable future but remains inaccessible (or unsustainable) in many of the countries that need it most. The resulting inadequate diagnostic capacity and lack of alternative reliable and inexpensive diagnostic tools remain threats to typhoid control.

On a more positive note, the long-awaited realization of opportunities for routine vaccination with TCV could ensure rapid reductions in typhoid burden, leading to elimination of typhoid fever in critical geographic areas if the vaccine performs as expected. Today, with these new vaccines, we are on the cusp of the turning point in typhoid history, but we must ensure that the time bought by vaccination leads to long-term investments in sustainable WASH improvements so that typhoid becomes of only historical interest.

## Notes

***Supplement sponsorship.*** This article is part of the supplement “Surveillance for Enteric Fever in Asia Project,” sponsored by the Sabin Vaccine Institute.

***Potential conflicts of interest.*** A. B. E. is a staff member of the WHO working on enteric fever. A. J. P. reports grants from the Bill & Melinda Gates Foundation, Wellcome Trust, National Institute for Health Research Oxford Biomedical Research Centre, Okairos, and Pfizer. A. J. P. is also the Chair of the UK Department of Health’s Joint Committee on Vaccination and Immunisation and the European Medicines Agency scientific advisory group on vaccines, and is a member of the WHO’s Strategic Advisory Group of Experts. All authors have submitted the ICMJE Form for Disclosure of Potential Conflicts of Interest. Conflicts that the editors consider relevant to the content of the manuscript have been disclosed.
